# Impact of early versus late parenteral nutrition on morphological and molecular markers of atrophy and autophagy in skeletal muscle of critically ill patients

**DOI:** 10.1186/cc12194

**Published:** 2013-03-19

**Authors:** I Vanhorebeek, MP Casaer, F Güiza, S Derde, I Derese, PJ Wouters, Y Debaveye, J Gunst, G Hermans, G Van den Berghe

**Affiliations:** 1 KU Leuven, Belgium

## Introduction

Muscle weakness of critical illness is associated with prolonged dependency on ventilatory support and delayed rehabilitation. Muscle wasting related to poor nutrition has long been considered a major determinant, whereas the importance of myofiber integrity only recently emerged [1-4]. We hypothesized that nutrient restriction early during illness aggravates atrophy while preserving myofiber integrity by activating the crucial cellular quality control pathway autophagy. The latter could be important to preserve muscle function.

## Methods

Critically ill patients (*n = *122) were randomized to early (early-PN) or late (late-PN) initiation of parenteral nutrition to complete failing enteral nutrition, while maintaining normoglycemia (80 to 110 mg/ dl) with insulin, in the EPaNIC study [5]. Vastus lateralis biopsies were harvested after 1 week and compared with matched controls (*n = *20).

## Results

As compared with controls, muscle from critically ill patients showed reduced myofiber density, a shift to smaller (especially type I) myofibers, lower myosin and actin mRNA, upregulated mRNA of the ubiquitin ligases muscle-ring-finger-1 and atrogin-1, a small increase in the autophagosome formation marker LC3-II/LC3-I, and increases in the autophagic substrates ubiquitin and p62 (all *P *≤0.006). Late-PN, resulting in a larger caloric deficit than early-PN, had no substantial impact on atrophy markers. In contrast, late-PN increased LC3-II/LC3-I (*P *= 0.02), which coincided with less accumulation of ubiquitinated proteins/aggregates (*P *= 0.05). Fewer patients on late-PN developed muscle weakness as compared with early-PN (42% vs. 68%, *P *= 0.05). In multivariable analysis, a lower LC3-II/LC3-I ratio (*P *= 0.05) and higher myofiber density (*P *= 0.04) were independently associated with muscle weakness.

## Conclusion

Early-PN did not counteract muscle atrophy whereas it suppressed autophagy and aggravated weakness. Statistically, muscle weakness was not explained by atrophy or wasting but rather by impaired autophagy and preservation of muscle density. Thus, tolerating nutrient restriction early during critical illness may preserve myofiber integrity by activating autophagy.
